# The 7th International Leh Symposium: Lungs at High Altitude: Molecular Mechanisms and Therapeutics of Hypoxic Lung

**DOI:** 10.1002/pul2.70023

**Published:** 2024-12-23

**Authors:** Rahul Kumar, Aastha Mishra, Arpana Vibhuti, Tashi Thinlas, Qadar Pasha

**Affiliations:** ^1^ Department of Medicine University of California San Francisco San Francisco California USA; ^2^ Lung Biology Center Zuckerberg San Francisco General Hospital San Francisco California USA; ^3^ Cardio Respiratory Disease Unit CSIR‐Institute of Genomics and Integrative Biology Delhi India; ^4^ Department of Biotechnology & Microbiology SRM University Delhi‐NCR Sonepat Sonipat India; ^5^ Department of Medicine Sonam Norboo Memorial Hospital Leh India; ^6^ Institute of Hypoxia Research Delhi India

The 7th International Leh Symposium on “Molecular Mechanisms and Therapeutics of Hypoxic Lung” from August 4 to 9, 2024, organized at the Mahabodhi International Meditation Centre in Saboo, Leh, Ladakh, India (Pictures 1), ensured the participation of a galaxy of national and international researchers, who explored the complex clinico‐physiological processes associated with high‐altitude adaptive and maladaptive mechanisms. The symposium provided a platform for sharing ideas and advancing knowledge to improve the quality of life and develop effective treatments for high‐altitude complications. Whereas, high‐altitude exposure is unavoidable for several reasons, such as over‐whelming tourism, endurance training, the presence of native high‐altitude populations, and military deployments.

**Picture 1:**
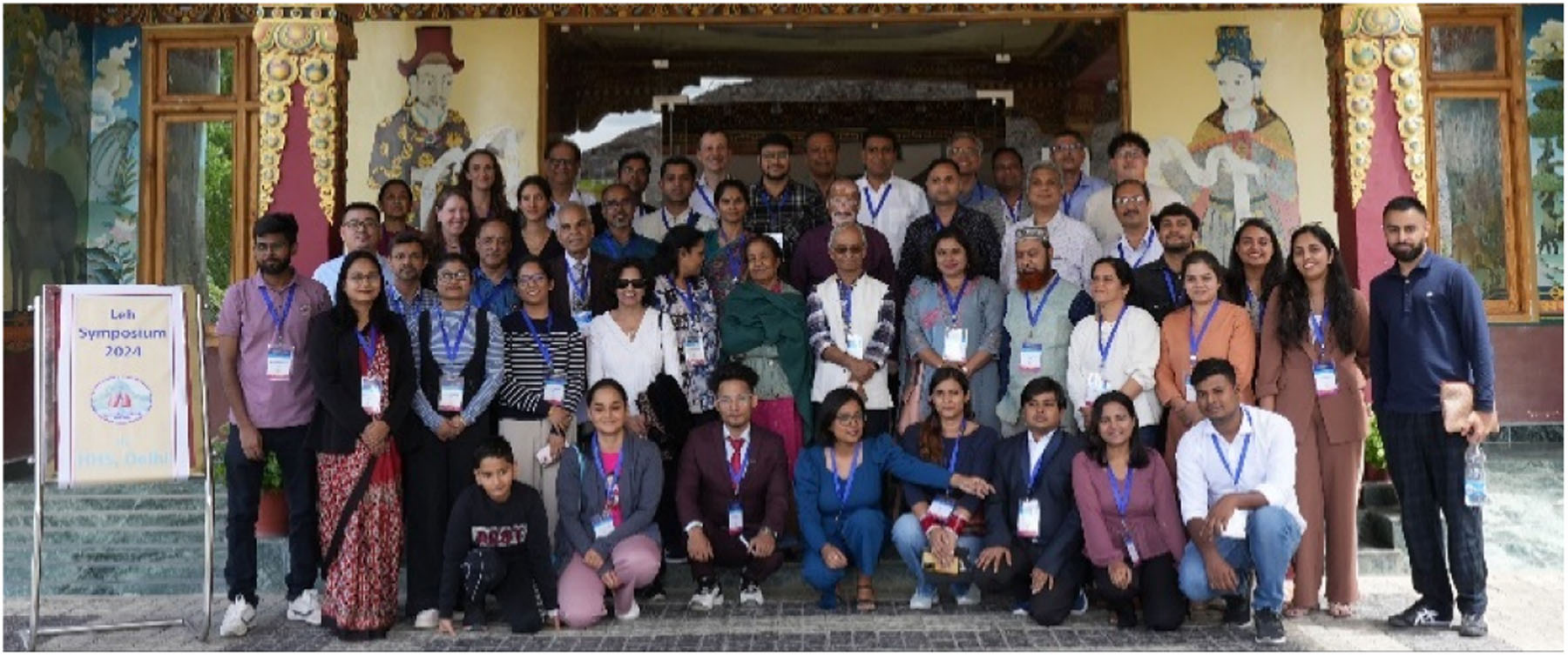
Conference attendees.

The inaugural session began with Nobel Laureate Professor Ratcliffe, who emphasized the role of HIF‐mediated pathways in adapting to high altitude and managing related vascular complications. He highlighted the critical role of genetic regulation in maintaining normal physiology under hypoxic conditions. The discussion on human metabolic adaptation to high‐altitude hypoxia concluded that metabolic responses to tissue hypoxia have important implications for health, both at altitude and in broader contexts like critically ill patients and pregnancy complications. Hypoxia triggers increased erythropoiesis through hypoxia‐inducible factors (HIFs), which regulate erythropoietin transcription. Evolutionary adaptations in high‐altitude populations—Tibetans, Ethiopians, and Andeans—reveal that Tibetans and Ethiopians maintain normal hemoglobin levels, while many Aymara and Quechua from the Andean highlands exhibit polycythemia. It was noted that NF‐κB interacts with HIFs to regulate erythropoiesis. The Aymara population expresses a distinct NFKB1 variant, which modulates inflammation by producing a truncated, nonfunctional NF‐κB peptide. This variant is associated with higher hemoglobin levels, lower leukocytes, and suppressed inflammation, marking a key genetic adaptation to high altitude in the Aymara people.

Immune Cell‐Mediated Inflammation and PH. “Immune Cell‐Mediated Inflammation play a significant role in the pathogenesis of and pulmonary hypertension (PH), with evidence linking autoimmune and infectious diseases to the development of PH. Inflammation can induce experimental PH, and perivascular inflammation is observed in human pulmonary arterial hypertension (PAH) and animal models. In both, inflammatory cell infiltrates—including mast cells, dendritic cells, fibroblasts, macrophages, B cells, and CD4/CD8 T cells. Experimental models have shown that anti‐inflammatory treatments, such as glucocorticoids and inhibitors of TGF‐β, IL‐6, IL‐21, SDF‐1, and macrophages, can reduce inflammation and PH symptoms. However, these results have not yet translated into clinical benefits for patients with PH, except in rare cases like lupus‐associated disease. Studies using *Schistosoma* and hypoxia‐induced PH models have provided valuable insights into immune cell involvement, but the causal relationship between inflammation and PH remains unclear. Whether inflammation is a trigger, amplifier, or merely an epiphenomenon of the disease is still under investigation. Systematic studies using hypoxia and *Schistosoma* or a combination of models could shed light on innate‐adaptive immunity crosstalk contributing to vascular disease. Presenters also drew parallels between PH and cancer, highlighting shared hypoxia‐related mechanisms. This ‘cancer hypothesis’ of PAH stems from the observation that PH involves an imbalance of cell proliferation and death, similar to neoplastic processes. Investigators have expanded this hypothesis to include the role of Hypoxia Inducible Factor (HIF‐1α), a key regulator of anaerobic glycolysis in both normal and cancer cells. Tumor microenvironments, such as those in hepatocellular carcinoma, show immune cells with metabolic changes that promote survival under hypoxic conditions. A new mechanism for lung cancer‐associated pulmonary hypertension (LC‐PH) was proposed, involving tumor‐associated macrophages and vascular remodeling.”

The Leh Symposium underscored the inseparable link between genetics, epigenetics, and high‐altitude adaptation, with experts focusing on the relevance of EPAS1 and EGLN1 variants. These genetic markers, identified in high‐altitude populations such as Tibetans, Ethiopians, and Andeans, enhance survival in hypoxic conditions. Unique adaptations, like those in the Aymara people, highlight the role of erythropoiesis and inflammation in high‐altitude survival. The discussion also extended to circulating cell‐free DNA (cfDNA) as a promising noninvasive biomarker for high‐altitude disorders. Elevated cfDNA levels were observed in severe cases of high‐altitude pulmonary edema (HAPE), suggesting its potential in diagnosis and monitoring. Similarly, exploring telomere system dynamics was presented as a new frontier in understanding and managing high‐altitude health challenges. Presentations further delved into how epigenetic regulation and genetic changes observed in highlanders support physiological and metabolic adaptations to hypoxia, crucial for maintaining oxygen delivery and overall health. However, these adaptations can impact cellular energetics, with broader implications for human health. The roles of telomeres, telomerase, and tankyrase (TNKS)—crucial in cancer, ageing, endurance, and respiratory diseases—were emphasized as equally significant in high‐altitude contexts, offering new approaches to managing health challenges in such environments.

Among other pulmonary diseases, Chronic obstructive pulmonary disease (COPD) and respiratory conditions like silicosis/anthracosis are significant health challenges, particularly in regions such as Ladakh, where environmental and socioeconomic factors exacerbate their prevalence. COPD, affecting millions globally, is fueled by smoking, biomass fuel exposure, and limited diagnostic access. In Ladakh, the high altitude, coupled with dust and biomass fuel use, contributes to a high prevalence of anthracosis, a condition often mistaken for COPD due to its similar clinical presentation and needs serious targeted attention and interventions. In parallel, anemia remains a critical concern in high‐altitude populations, with recent data suggesting that hypoxia‐related correction factors might lead to inflated prevalence estimates. Localized research highlights a discrepancy between anemia prevalence before and after applying such adjustment factors, indicating the need for tailored population‐specific diagnostic approaches for newborns in these regions.”.

Apart from the pulmonary, the neurological system is equally relevant. The illnesses include acute mountain sickness (AMS), high altitude cerebral edema (HACE), and insignificantly stroke. Experts highlighted the evolution of oxygen‐dependent organisms and how rising atmospheric oxygen led to the human brain's reliance on it for continuous activity. Despite oxygen's essential role in the CNS, its paradoxical nature—both vital and harmful—was emphasized, with its quantum properties offering new insights into brain disease pathophysiology. Regional variations in HA‐related conditions were influenced by factors like geographic location, altitude, and exposure duration. Hypoxia‐induced hypercoagulability and other unique risk factors like thrombosis/thromboembolism (Choose the correct term) highlight the need for tailored preventive strategies, especially in younger people and those with cardiovascular issues.

Enhancing High‐Altitude Healthcare with artificial intelligence (AI) was another relevant topic. Its role in today's scientific world is impeccable, as in Advanced Imaging. This session explored the transformative potential of AI in high‐altitude healthcare. AI tools are expected to significantly improve disease diagnosis and understanding of high‐altitude conditions, offering timely clinical decision support and enhanced detection. These technological advancements address both moral and logistical challenges in managing high‐altitude health crises.

Therapeutics at altitudes has remained a burning topic since its inception. Various drugs have been tried and tested; among them, acetazolamide and dexamethasone found a distinct place. Sex‐specific effects of Dexamethasone indicated it might alleviate high‐altitude disorders by inhibiting early inflammation. Among the very interesting explorative studies, Beetroot juice was shown to improve acclimatization, and Quercetin outperformed acetazolamide in mitigating oxidative stress. Ormeloxifene showed promise for treating pulmonary hypertension by enhancing estradiol synthesis and reducing inflammation. HMGB3's dual role in cardiac ischemia and repair was also noted, alongside estrogen receptor alpha (Erα) involvement in pulmonary adaptation to high altitudes. Moreover, developing targeted anti‐inflammatory therapies for early intervention in PH was echoed.

Young Investigators' and Posters' Presentations and Awards were a delight to attend. At the Young Investigator Award session, researchers presented novel findings on high‐altitude physiology. Key insights included unique adaptations in Andean and Tibetan populations, correlations between hemoglobin levels and exercise capacity in pulmonary arteriovenous malformations, and genetic links to polycythemia among Tibetans. Elevated cfDNA levels and oxidative stress were noted in HAPE patients. Dr. Taylor Shay Harman from Syracuse University received the Qadar Pasha‐Young Investigator Award, including a certificate and Rs. 25,000 for her exceptional research (Pictures 2). Posters highlighted the genetic advancements such as genetic markers for cardiac channelopathies, benefits of remote ischemic conditioning in IPF, cfDNA as a HAPE marker, and the role of genetic variants in viral infection susceptibility. Other studies explored how beetroot juice improves acclimatization, quercetin outperforms acetazolamide in mitigating oxidative stress, and yoga reduces oxidative stress. The Best Poster Award on cfDNA went to Mr. Manzoor Ali from CSIR‐Institute of Genomics and Integrative Biology, earning a certificate and Rs. 7000. Three additional outstanding posters were recognized, each awarded a certificate and Rs. 5000.

**Picture 2:**
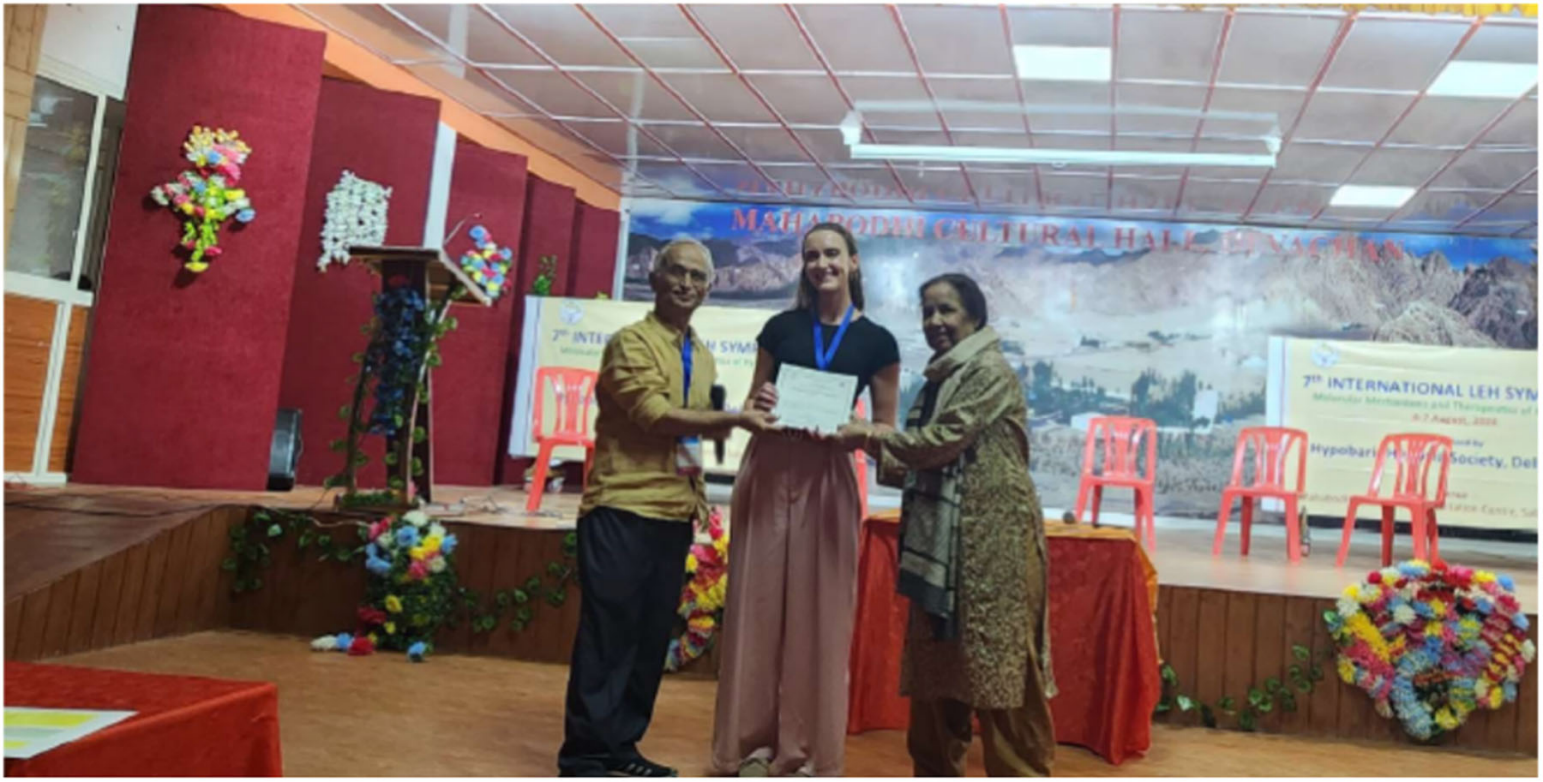
Qadar Pasha‐Young Investigator award.

Finally, the Panel Discussions, introduced for the first time, on a high demand, deserve a space. The session on ‘Evidence‐Based Traditional Medicine for High‐Altitude Wellness' discussed the scientific benefits of yoga in high‐altitude settings, highlighting its role in reducing ROS and increasing NO levels, which enhance endurance and improve endothelial function. Yoga lowered pulse rates through parasympathetic activation and induced a marginal increase in oxygen saturation, suggesting a shift to anaerobic metabolism. These physiological effects may improve cognitive function and mental relaxation, crucial for managing high‐altitude conditions like HACE and cognitive impairments. Additionally, Yoga's ability to reduce cortisol and oxidative stress was noted, potentially alleviating DNA damage‐induced inflammation. The session on “Managing Clinical Hypoxia at High Altitude” focused on effective strategies for managing AMS and associated comorbid conditions. Key points included the necessity for proactive management of individuals with pre‐existing conditions, such as high blood pressure, and the importance of tailored recommendations to mitigate high‐altitude risks. The experts emphasized the need for comprehensive care and preparation to ensure safe high‐altitude experiences; they suggested that an elevated 24‐h mean arterial pressure (MAP) at high altitudes was not clinically significant and did not correlate with adverse outcomes, even in those with hypertension.

Overall outcome and take‐home message from the 7th Int'l Leh Symposium: It promoted valuable knowledge‐sharing and international collaboration on high‐altitude health issues, including AMS, HAPE, PH and HACE, and their impact on both sojourners and residents. Key discussions addressed the potential benefits of Dexamethasone, Acetazolamide, quercetin, and beetroot supplements alongside emerging research on inflammation‐targeted drugs and telomere length regulation. The primary takeaway emphasized translating these findings into practical healthcare solutions to enhance health outcomes for individuals in high‐altitude environments. The Valediction concluded with a decisive focus on creating comprehensive guidelines for visiting sojourners as a precautionary healthcare measure. This initiative received overwhelming support, and a team of experts will develop these guidelines.

**Acknowledgments:** Heartfelt thanks to all delegates, PhD scholars, and young scientists for their participation, as well as DST, DBT, CSIR, and the pharmaceutical industry for sponsorship. Special thanks to SNM Hospital, Venerable Guruji Shri Bhikkhu Sanghasena, Prof G.L. Sharma and volunteers for their invaluable support and contributions to the Symposium's success. We appreciate the Pulmonary Vascular Research Institute (PVRI), UK and the journal *Pulmonary Circulation* for publishing the abstracts and for always being with us.

MEETING ABSTRACT

Open Access

**Abstracts from the 7** ^**th**^ **International Leh Symposium, India**

*Molecular Mechanisms and Therapeutics of Hypoxic Lung*

**A001 SELECTIVE ESTROGEN RECEPTOR MODULATION CONFERS PROTECTION AGAINST PULMONARY HYPERTENSION**

Adam Olaitan Abdulkareem, Kumaravelu Jgavelu, Kashif Hanif

Division of Pharmacology, CSIR‐Central Drug Research Institute, Lucknow, India

Background: The protective effect of 17β‐estradiol is well‐known in pulmonary hypertension. However, estrogen‐based therapy may potentially increase the risk of breast cancer, necessitating a search for novel drugs. This study, therefore, investigated the ameliorative effects of a selective estrogen receptor modulator, ormeloxifene, in pulmonary hypertension. Materials and Methods: Female (ovary‐intact or ovariectomised) rats received monocrotaline (60 mg/kg, once, subcutaneously), with or without ormeloxifene treatment (2.5 mg/kg, orally) for 4 weeks. Results: Monocrotaline decreased plasma 17β‐estradiol and uterine weight in ovary‐intact rats. Further, monocrotaline altered 17βHSD1 and 17βHSD2 expressions and reduced estrogen receptors α and β, increasing right ventricular pressure, proliferation, inflammation, oxidative stress, endothelial dysfunction, mitochondrial dysfunction, and vascular remodeling in female and male rats, with worsened conditions in ovariectomised rats. Ormeloxifene attenuated both monocrotaline‐induced effects by improving pulmonary 17β‐estradiol synthesis. Furthermore, ormeloxifene decreased cardiac hypertrophy and right ventricular remodeling induced by monocrotaline. Conclusion: This study demonstrated that ormeloxifene promoted pulmonary 17β‐estradiol synthesis, alleviated inflammation, improved the NOX4/HO1/Nrf/PPARγ/PGC‐1α axis, and attenuated pulmonary hypertension. It is evidently safe at tested concentrations and may be effectively repurposed for pulmonary hypertension treatment.

**A002 PROFILE OF CARDIOMYOPATHIES FROM JAMMU & KASHMIR AND LADAKH**

Ajay Bahl, Vanya Vaidya, Anupam Mittal, Maryada Sharma, Madhu Khullar

Department of Cardiology, Postgraduate Institute of Medical Education and Research, Chandigarh, India

Background: Most cardiomyopathies are genetic disorders. Variation in founder mutation in different regions may result in some differences in clinical profiles. There is limited data on profile of cardiomyopathies in patients from Jammu & Kashmir and Ladakh. The profile of cardiomyopathies in patients from Jammu and Kashmir & Ladakh studied. Materials and Methods: All patients in the cardiomyopathy cohort from Jammu & Kashmir and Ladakh of a tertiary care center were analysed. Of the 1333 patients, 32 (2.4%) were from these areas. Results: Twenty‐one patients presented with hypertrophic cardiomyopathy (HCM) like picture 6 had dilated cardiomyopathy (DCM), and 5 had restrictive cardiomyopathy (RCM). Twelve (37.5%) patients were females. Mean age was 41.8 ± 18.8 years (range 10–81 years). One patient with ventricular hypertrophy and three patients with RCM‐like presentation had AL amyloidosis. One DCM patient had eosinophilic heart disease and one had left ventricular noncompaction. Next generation sequencing was carried out in six patients. Mutations were detected in two patients. One HCM patient had a *MyBPC3* variant and one RCM patient had a *FLNC* variant. Endomyocardial biopsy was carried out in six patients. Of these, three had AL amyloidosis while three had nonspecific findings. Six (18.8%) patients died during follow‐up. Four had AL amyloidosis and two had HCM. Al amyloidosis had dismal outcomes due to late diagnosis, with all four patients dying during follow‐up. Conclusion: Endomyocardial biopsy was useful in establishing diagnosis, had good diagnostic value in uncertain cases. Genotyping identified mutations in one‐third of patients with HCM or RCM.

**A0003 HUMAN METABOLIC ADAPTATION TO HIGH‐ALTITUDE HYPOXIA**

Andrew Murray

Department of Physiology, Development and Neuroscience, School of the Biological Sciences, University of Cambridge, UK

Background: Ascent to high altitude is accompanied by physiological responses that mitigate the challenge of hypobaric hypoxia, maintaining arterial blood oxygen content and convective oxygen delivery. At the tissues, oxygen utilization also adjusts over time in response to altered oxygen availability through metabolic alterations that typically suppress mitochondrial respiratory capacity and fatty acid oxidation. In lowlanders, acclimatization can nevertheless result in compromised cellular energetics, yet studies in human populations of highland ancestry have revealed physiological traits, underpinned by genetic variants, that have undergone selection and allow people to live, work and reproduce at high altitude. Of note, placental metabolic adaptations form a vital component of an integrated response that supports healthy pregnancies in highlander populations at altitude. Metabolic responses to tissue hypoxia have important implications for human health both at altitude and in human disease more generally, being associated with outcomes in critically ill patients and common complications of pregnancy.

**A004 DIAGNOSTIC IMAGING AT HIGH ALTITUDE‐ OVERCOMING STRATOSPHERIC CHALLENGES WITH PLAUSIBLE SOLUTIONS**

Anjali Agrawal

Senior Consultant and Delhi Head, Teleradiology Solutions, Delhi, India

Background: Radiology, by virtue of its sophisticated machinery, cumbersome installations in shielded rooms, and regular technical supervision, remains confined to urban areas. Materials: Technological advancements, particularly in point‐of‐care imaging, have made its penetration into rural and relatively hostile terrains possible. Unfortunately, technologists, particularly radiologists, who are required to perform and interpret these exams remain in short supply and are concentrated in urban landscapes for lifestyle and professional reasons. Results: Teleradiology has been a game‐changer in healthcare by partially improving access and availability to expert radiological opinion beyond geographical boundaries and narrowing the wide demand‐supply gap. Now, with artificially intelligent tools on‐site or on the cloud, one can expect a timely clinical decision support on‐site, with improved detection, quantification as well as prediction. Conclusion: The AI tools would perhaps objectivize the decision‐making to transfer an ailing Sherpa to a lower altitude while easing the moral dilemma of costing a man his livelihood.

**A005 TELOMERE DYNAMICS IN HYPOBARIC HYPOXIA PATHOPHYSIOLOGY**

Arpana Vibhuti^1^, Rohit Kumar^1, 2^, Arun Paulose^1, 2^, Aastha Mishra^2^,

Qadar Pasha^2, 3^

^1^Department of Biotechnology and Microbiology, SRM University Delhi‐NCR, Sonepat, India

^2^Genomics and Genome Biology Unit, CSIR‐Institute of Genomics and Integrative Biology, Delhi, India

^3^Institute of Hypoxia Research, New Delhi, India

Background: Telomeres are a novel and promising target to explicate the phenomena of adaptation/maladaptation at high altitude (HA). The endurance of an individual may be prognosed in advance by studying the telomeres of the people before they visit high altitudes. Hypobaric hypoxia at HA generates reactive oxygen species (ROS) that can damage telomeres and disturb normal physiological processes. The telomere complex comprises multiple proteins, of which tankyrase (TNKS) is actively involved in DNA damage repairs. Materials: We investigated this system in three groups: the healthy sojourners named HAPE‐resistant (HAPR‐r), healthy natives (HLs) and the HA Pulmonary Edema patients (HAPE‐p). Results: The telomere length was shorter in HAPE‐p compared to HAPE‐r (*p* = 0.03) and HLs (P = 4.25E‐4). The telomerase activity was significantly higher in HAPE‐p compared to both HAPE‐r (*p* = 0.01) and HLs (*p* = 0.001). HAPE‐p had the lowest TNKS levels (0.186 ± 0.031 ng/μl) and the highest telomerase activity (0.0268 amoles/μl). The expression was upregulated by 9.27 folds in HAPE‐p (P = 1.01E‐06) and downregulated in HLs by 3.3 folds (*p* = 0.02). The findings indicated the association of telomere, telomerase and TNKS with hypoxia‐induced sicknesses/acclimatization. To understand the roles of telomeres, understanding their regulation, such as through epigenetics, is critical. The epigenetic modifications promote red blood cell synthesis and more effective oxygen utilization, essential for survival in HA settings. Conclusion: Thus, correlations between the telomere system and methylation could provide greater insight into HA sickness. Moreover, these correlations may help us better understand the intricate biological reactions to hypoxia and create preventative and therapeutic measures.

**A006 IMMUNE CASCADE IN INFLAMMATORY PULMONARY VASCULAR DISEASE INCLUDING** ***SCHISTOSOMA*** **AND HYPOXIC PULMONARY HYPERTENSION**

Brian B. Graham^1,2^, Rahul Kumar^1,2^, Qadar Pasha^3,4^

^1^Department of Medicine, University of California San Francisco, San Francisco, CA, USA

^2^Lung Biology Center, Division of Pulmonary and Critical Care Medicine, Zuckerberg San Francisco General Hospital, San Francisco, CA, USA

^3^Genomics and Genome Biology Unit, CSIR‐Institute of Genomics and Integrative Biology, Delhi, India

^4^Institute for Hypoxia Research, Delhi, India

Background: Inflammation is a major driver of many forms of clinical and experimental pulmonary hypertension (PH). Materials and Methods: Our collaborative group has studied pulmonary vascular inflammation in the context of *Schistosoma*‐ and hypoxia‐induced disease in animal models and clinical specimens, to discover pathologic phenotypes of the host. Results: In *Schistosoma*, embolization of parasite eggs to the lungs causes a cascade of Type 2 inflammation leading to TGF‐beta activation, which may benefit the host immune system by quelling excessive inflammation. In hypoxia, there is a sterile recruitment of monocytes to the lung parenchyma, which results in thrombospondin‐1 expression and TGF‐beta activation. We thus find a convergence of varied proximate immune signals on TGF‐beta signaling, a pathway shared with forms of familial PH driven by mutations in the TGF‐beta family, and successfully targeted by the recently developed sotatercept. One important area of uncertainty is how the host inflammation phenotype changes over time, in the setting of either persistent or ceased immune stimulus, and if the immune system continues to drive ongoing vascular disease or may contribute to disease resolution. In *Schistosoma*‐induced PH in mice, there is early evidence that repeated exposure causes persistent late disease, by which time the inflammation has largely subsided. Conclusion: Thus, targeting inflammation may be particularly effective in preventing the development of PH in at‐risk individuals but could be found to be less effective in reversing late established disease.

**A007 BEYOND THE PEAKS: FATALITIES IN THE SWISS MOUNTAINS, THEIR PSYCHOLOGICAL IMPACT AND PEER SUPPORT FOR MOUNTAIN RESCUERS**

Corinna A. Schön

Institute of Forensic Medicine, Kantonsspital St. Gallen, Switzerland

Medical Chief, Alpine Rescue Switzerland

President of the Swiss Society of Mountain Medicine, Switzerland

Background: Switzerland has a well‐developed rescue system that does not only work in metropolitan areas. Even in less densely populated rural areas and even in the mountains, sick and injured people can often be quickly rescued and/or given medical care thanks to several air rescue organizations, the volunteers of Alpine Rescue Switzerland, and the support of medically trained lay rescuers. The number of mountain rescue operations has increased continuously in recent years, which, among other things, can be explained by the increase in tourism numbers, changes in climate and an increased interest in alpine sports.

Especially in the mountains, rescue personnel often encounter difficult events. These include patients with serious injuries, events involving multiple victims or even fatalities. In addition to objective dangers (avalanches, rockfall, storms, etc.) in the high mountains, mountain rescuers are therefore additionally exposed to intense situations that come with an increased risk of psychological stress, including the development of psychiatric complications such as posttraumatic disorder. This was proven in a study among rescuers from Alpine Rescue Switzerland. Because of these findings, a model for early support by peers to prevent secondary diseases was developed. This project will be presented here.

**A008 GENDER DIFFERENCE AND POLYCYTHEMIA ASSOCIATED WITH POLYMORPHISM IN RS13419896 AND RS2790859 AMONG TIBETAN HIGHLANDERS LIVING IN MUSTANG, NEPAL**

Hiroaki Arima^1^, Sweta Koirala^2^, Takayuki Nishimura^3^, Yoshiki Yasukochi^4^, Taro Yamamoto^1^

^1^Institute of Tropical Medicine, Nagasaki University, Nagasaki, Japan

^2^Nepal Development Society, Pokhara, Nepal

^3^Department of Human Science, Kyushu University, Fukuoka, Japan

^4^Department of Genome Analysis, Institute of Biomedical Science, Kansai Medical University, Osaka, Japan

Background: This study investigates the association between genetic polymorphisms and the incidence of lifestyle‐related diseases and polycythemia among Tibetan highlanders in Mustang, Nepal, who have adapted to hypoxic environments by avoiding increased hemoglobin concentration and polycythemia. Recent concerns suggest that modernization and dietary changes may increase the risk of these diseases. Methods and Materials: The study aims to elucidate whether polymorphisms in hypoxic adaptation genes (*EPAS1* and *EGLN1*) influence disease incidence and hemoglobin dynamics with age. We involved compiling health data from 2017 for 168 participants (78 males, 90 females) in Mustang, including the prevalence of obesity, hypertension, diabetes, hypoxemia, and polycythemia. DNA was extracted, and real‐time PCR was used to obtain polymorphic data for rs13419896 (*EPAS1*), rs12619696 (*EPAS1*), and rs2790859 (*EGLN1*). Statistical analysis assessed associations between health outcomes and these genetic polymorphisms. Results: The results indicated significant gender differences in disease prevalence, particularly polycythemia (*p* = 0.0009). Significant gender differences were also observed in genotype frequencies for rs13419896 and rs2790859. Among females, those with the Tibetan‐adapted rs2790859 genotype had a significantly lower incidence of polycythemia (*p* = 0.0016) and lower hemoglobin levels compared to the non‐adapted genotype (*p* = 0.0083). Conclusion: Our findings suggest gender differences in hypoxic adaptation gene frequencies exist in the Mustang population, with the rs2790859 polymorphism associated with polycythemia among Tibetan highlanders. As ethnic intermixing and dietary changes continue in Mustang, the prevalence of nonadaptive alleles may increase, potentially making polycythemia a modern disease in the region.

**A009 ALTERNATIVELY SPLICED** ***NFKB1*** **CAUSES ELEVATED HEMOGLOBIN LEVELS IN ANDEAN AYMARAS**

Jihyun Song^1^, Seonggyun Han^2^, Ricardo Amaru^3^, Lucie Lanikova^4^, Teddy Quispe^3^, Dongwook Kim^2^, Jacob E. Crawford^5^, Younghee Lee^6^ and Josef T. Prchal^1^

^1^Division of Hematology and Hematologic Malignancies, Huntsman Cancer Institute, University of Utah, Salt Lake City, UT, 84112, USA

^2^Department of Biomedical Informatics, School of Medicine, University of Utah, Salt Lake City, UT, 84112, USA

^3^Cell Biology Unit, University of San Andres, National Academy of Sciences, La Paz, Bolivia

^4^Department of Cell and Developmental Biology, Institute of Molecular Genetics of the Czech Academy of Sciences, Prague, Czech Republic

^5^Verily Life Sciences, South San Francisco, CA, 94080, USA

^6^College of Veterinary Medicine and Research Institute for Veterinary Science, Seoul National University, Seoul, Korea 08826, Republic of Korea

Background: Three populations—Tibetans, Ethiopians, and Andeans—reside at high altitudes and exhibit unique genetic adaptations to high‐altitude hypoxia. In a hypoxic environment, Tibetans and Ethiopians maintain hemoglobin (Hb) levels similar to those at sea level, whereas Andeans exhibit higher Hb levels compared to Europeans at equivalent altitudes. Previous studies have identified two genetic variants within the hypoxia‐inducible factor (HIF) pathway—*EPAS1* (HIF‐2α) and *EGLN1* (prolyl hydroxylase 2)—that correlate with the low Hb levels observed in Tibetans at high altitude (Science 2010, Nat Gen 2014). Our whole genome sequencing analysis of the Aymara (Am J Hum Genet. 2017) discovered significant selection signals in the *BRINP3*, *NOS2*, and *TBX5* genes associated with cardiovascular function and development. However, these findings did not account for the elevated Hb levels observed in the Aymara. Materials: We analysed the whole transcriptome of granulocytes to understand the genetic adaptations contributing to high Hb levels in the Aymara population living at high altitudes. Peripheral blood samples from Aymara and European individuals were collected at La Paz in Bolivia (4,000 m). Results: Using DESeq. 2 and rMATS, we identified 2,601 differentially expressed genes and 1,922 differentially spliced transcripts in Aymaras compared to Europeans, associated with immune, inflammatory, and hypoxia‐related pathways. We then evaluated cis‐genetic regulators, focusing on expression quantitative trait loci (eQTLs) and splicing quantitative trait loci (sQTLs). Within the *NFKB1* gene, which is essential for suppressing inflammation and activating HIFs, we discovered novel alternatively spliced transcripts (AS‐*NFKB1*) with skipped exons 4 or 5, or both. AS‐*NFKB1* transcripts missing exon 4 or both exons 4 and 5 failed to produce a functional protein. In contrast, AS‐NFKB1 lacking only exon 5 produced a protein that was improperly processed and unable to translocate to the nucleus, resulting in a complete or partial loss of NFKB1's canonical function. AS‐*NFKB1* transcript levels correlated with 5 sQTLs and were enriched in Aymaras compared to other populations. Among these 5 sQTLs, rs230511 was the most selected single‐nucleotide polymorphism (SNP) in Aymaras. The T allele of rs230511, enriched in the Aymara population, was positively correlated with elevated Hb levels and increased expression of NF‐kB‐regulated inflammatory genes, including interferon gamma and interleukin 6. Although augmented inflammation suppresses erythropoiesis by upregulating hepcidin, AS‐*NFKB1* was associated with high Hb. AS‐*NFKB1* correlated with a significant upregulation of HIF‐regulated genes, principal regulators of augmented erythropoiesis, which explains Aymara's erythrocytosis. These findings suggest that increased HIF‐transcriptional activity in Aymara erythrocytosis overcomes the suppression of erythropoiesis by increased inflammation mediated by AS‐NFKB1. We have found genetic markers linked to the elevated Hb levels observed in Aymaras, along with their molecular mechanisms. Conclusion: Our findings establish a connection between Aymara‐enriched *NFKB1* SNPs and AS‐*NFKB1*, indicating their association with elevated Hb levels and increased inflammation. However, the evolutionary advantage of increased inflammation in Aymaras remains to be determined.

**A010 RESPIRATORY TRACT INFECTIONS AS POTENTIAL RISK FACTORS FOR HIGH ALTITUDE PULMONARY EDEMA DEVELOPMENT**

Kanika Singh^1,2^, Krishna Kumar G.^1^, Manzoor Ali^1,2^, Stanzen Rabyang^3^,

Tashi Thinlas^3^, Aastha Mishra^1,2^

^1^Genomics and Genome Biology Unit, CSIR‐Institute of Genomics and Integrative Biology, Delhi, India

^2^Academy of Scientific and Innovative Research (AcSIR), Ghaziabad, India

^3^Department of Medicine, Sonam Norboo Memorial Hospital, Leh, India

Background: High altitude (HA, altitude > 2500 m above sea level) presents a unique set of environmental conditions that significantly impact human health. As altitude increases, atmospheric pressure decreases, leading to lower oxygen levels, extreme temperatures, and intensified solar radiation. These extreme environmental conditions may affect the respiratory health of unacclimatized and susceptible sojourners, which may lead to noncardiogenic pulmonary edema, known as high altitude pulmonary edema (HAPE). Sojourners with mild respiratory infection are already in a state of respiratory compromise, which, upon exposure to the hypobaric hypoxic condition at HA, may worsen further. Respiratory tract infections (RTI), as an important risk factor in enhancing HAPE susceptibility in sojourners, are still unexplored in HA settings. Materials: Microbiome profiling in nasal swabs and sputum samples of HAPE patients and healthy HA sojourners can establish RTI as one of the risk factors for HAPE susceptibility. HAPE patients are further segregated into mild, moderate, and severe categories based on clinical symptoms and X‐ray imaging. Microbiome profiling approaches such as shotgun sequencing for metagenomics can identify microbial diversity, community structure, and pathogen prevalence. Integration of metagenomics with RNA sequencing or metatranscriptomics can simultaneously interrogate the interindividual host responses, antibiotic resistance, and mechanistic insights into the disease. Such integration holds the capacity to detect all potential pathogens including bacteria, viruses, fungi and parasites present in upper and lower respiratory tract via the nasal swab and sputum samples, respectively. Conclusion: Therefore, correlating microbiome data with clinical parameters can further infer a higher prevalence of microbial load with disease severity. Such analysis can help identify better prevention strategies, paving the way for specific medicine practices and mitigating the health risks associated with HA travel and habitation.

**A011 ROLE OF BEETROOT JUICE SUPPLEMENTATION ON PHYSIOLOGICAL RESPONSE OF HEALTHY MALES EXPOSED TO HIGH ALTITUDE**

Karishma Dohare, Subhasis Bose, Divya Singh, Praveen Vats

Defence Institute of Physiology and Allied Sciences, Defence Research and Development Organisation, Timarpur, Delhi, India

Background: High altitude hypoxia is the most common threat associated with high altitude (HA) ascend. People ascending to HA face several challenges, including hyperventilation, vasoconstriction and decreased oxygen saturation. Beetroot, rich in anti‐oxidants, nitrate, minerals and vitamins, has emerged as a superfood in recent years. It has anti‐inflammatory, antioxidant and vasodilatory functions. Materials and Methods: Therefore, in the present study, we investigated the effect of beetroot juice (BRJ) supplementation on human physiological response to exposure to HA. Healthy human males participated in this study, randomly assigned to BRJ (*n* = 63) and control group (*n* = 40). All measurements were done at sea level and then at HA before and after supplementation. BRJ group consumed 200 mL juice/day for 15 days. Results: We found that BRJ supplementation increases the salivary and plasma nitrite at HA in comparison to control group. We observed substantial changes in physiological response on BRJ supplementation. Blood pressure and heart rate decreases significantly in BRJ group while the control group does not show marked improvement. We also observed improvement in peripheral oxygen saturation and a lesser decrease in VO_2_ max in supplemented group. Hematological and biochemical analysis reveals an increment in hemoglobin and iron levels in BRJ group. It has been well‐established that increase in Hb, iron and nitrite are linked with acclimatization process at HA. Conclusion: The overall observations made during the study suggest that the implementation of beetroot may have the potential to mitigate the adverse effects of HA exposure.

**1012 IMPACT OF YOGA ON STRESS: EXPLORING ITS ROLE IN DNA DAMAGE RESPONSE AND INFLAMMATION**

Khushboo Arya^1,2^, N.K. Manjunath^2^, Akshay Anand^3^, Sheetal Sharma^1^

^1^Department of Experimental Medicine and Biotechnology, Postgraduate Institute of Medical Education and Research, Chandigarh, India,

^2^Swami Vivekananda Yoga Anusandhana Samsthana, Bengaluru, India,

^3^Neuroscience Research Lab, Department of Neurology, Postgraduate Institute of Medical Education and Research, Chandigarh, India

Background: Inflammation through production of pro‐inflammatory cytokines is a known outcome of free radicals generation. Impact of such oxidative stress on endothelial function can be summarized into reduced bioavailability of nitric oxide (impairing vasodilation), increased endothelial permeability and enhanced expression of adhesion molecules leading to migration of immune cells into the vessel wall perpetuating inflammation. Resultant reduced oxygen availability at the cellular level causes hypoxia, metabolic shift to anaerobic glycolysis and activation of Hypoxia‐Inducible Factors (HIFs). Yoga, an adjunct therapy widely used today, known to bring down the hyperactivation of inflammatory markers, may affect cellular hypoxia by keeping stress molecules in check. In this study we aim to evaluate the difference between stress levels among groups that practice yoga compared to the group that has never practiced yoga. We also wanted to assess the effect of stress on DNA damage response in normal cells. Materials and Methods: Stress level of the subjects was evaluated using Cohen's perceived stress scale and molecular parameters followed by assessment of oxidative stress. Further, the levels of a DNA damage marker upon cortisol‐induced stress were checked in normal cells. Results: Yoga reduces cortisol levels among practitioners and may also impact oxidative stress levels. Interestingly, it was observed that cortisol induces DNA damage in normal human cells, a precursor for inflammation. Conclusion: These findings indicate that yoga may reduce inflammation induced by DNA damage, improving overall survival.

**A013 ENDMOTIF DIVERSITY IN CIRCULATING CELL‐FREE DNA UNDER HYPOBARIC HYPOXIC CONDITION OF HIGH‐ALTITUDE ENVIRONMENT**

Krishna Kumar G.^1^, Manzoor Ali^1,2^, Raushni Choudhary^1,2^, Kanika Singh^1,2^,

Stanzen Rabyang^3^, Tashi Thinlas^3^, Aastha Mishra ^1,2^

^1^Genomics and Genome Biology Unit, CSIR‐Institute of Genomics and Integrative Biology, Delhi, India

^2^Academy of Scientific and Innovative Research (AcSIR), Ghaziabad, India

^3^Department of Medicine, Sonam Norboo Memorial Hospital, Leh, India

Background: Extreme environmental conditions such as decreased barometric pressure, high ultraviolet radiation, cold, and low humidity are the characteristics of high altitude (HA). When sojourns rapidly ascend to HA without any proper precautions, they might suffer HA illnesses due to hypobaric hypoxia. One such HA illness is high altitude pulmonary edema (HAPE), whose pathogenesis involves hypoxic pulmonary vasoconstriction, which further leads to failure of capillary stress in the lungs, causing edema formation in alveoli. Circulating cell‐free (cf) DNA is the extracellular DNA found in various bodily fluids such as blood, urine, cerebrospinal fluid (CSF), and sweat. The cfDNA as a marker got its limelight in oncology and noninvasive prenatal testing (NIPT). The noninvasive nature of cfDNA and as a trove of tissue signatures make it a suitable marker to study any disease manifestation. Due to the nonrandom fragmentation patterns of cfDNA, certain unique motif features at the fragment end alter during stress exposure. Materials and Methods: To determine any diversity of motifs in HAPE patients, comparisons are made with the healthy sojourners who visited HA but did not develop HAPE and the native highlanders. Deep sequencing of cfDNA fragments is conducted to study the feature 4‐mer 5′ end motif and the Motif diversity score (MDS) measures the diversity of cfDNA motifs across the groups. Results: The higher MDS indicates an equal proportion of all motifs, and in the case of lower MDS, it indicates that few motifs have been enriched. Conclusion: Epigenetic events involved in HAPE pathophysiology can be identified using MDS integrated with other cfDNA features.

**A014 DUAL ROLE OF DAMAGE ASSOCIATED MOLECULAR PATTERN HMGB3 IN MYOCARDIAL ISCHEMIA**

Kumaravelu Jgavelu

CSIR‐ Central Drug Research Institute, Sector 10, Janakipuram Extension, Lucknow, India

Background: Myocardial ischemia and infarction are the number one cause of cardiovascular disease mortality. Cardiomyocyte death during ischemia leads to the loss of cardiac tissue and initiates a signaling cascade between the area at risk and the infarct zone of the myocardium. Here, we sought to determine the involvement of damage‐associated molecular patterns in high mobility group protein B3 (HMGB3) in myocardial ischemia and infarction. Materials and Methods: We used the left anterior descending coronary artery ligation model to study the involvement of HMGB3 in myocardial infarction. Results: Our results indicated the presence of HMGB3 at a low level under normal conditions, while myocardial injury caused a robust increase in HMGB3 levels in the heart. Further, intracardiac injection of mabHMGB3 improved cardiac function on Day 3 by downregulating HMGB3 levels. In contrast, injection of recombinant rat HMGB3 for 7 days during the adaptation phase of myocardial ischemia improved cardiac functional parameters by increasing regenerative protein family expression. Further, to mimic the disease condition, rat ventricle cardiomyocytes and fibroblasts were exposed to hypoxia; we observed a significant upregulation in the HMGB3, HIF1α, and Reg1α levels. Endothelial cells exposed to recombinant HMGB3 increased the tubule length. Further, the mitochondrial oxygen consumption rate was reduced with the acute induction of recombinant HMGB3 on cardiomyocytes and fibroblasts. HMGB3 plays a dual role during the progression of myocardial infarction. Conclusion: Clinically, post‐myocardial infarction HMGB3 induced sterile inflammation must be tightly controlled effectively as it plays both a pro‐inflammatory role and improves cardiac function during the cardiac remodeling phase.

**A0015 DOES BLOOD PRESSURE MATTER FOR TRAVELERS TO HIGH ALTITUDE?**

Linda E. Keyes, Greta Kreider Carlson, Elan Small, Diana Biggs, Andrew C. Burns, Tiana Linkus, Ilaria Ferrari, Lukas Sloan

Emergency Medicine, University of Colorado, USA

Background: Travelers to high altitude (HA) frequently worry about their blood pressure, especially those with underlying hypertension. The evidence driving clinical recommendations, however, is limited. Materials and Methods: We compared 24‐h ABP at low altitude (LA) versus HA in tourists and examined related outcomes. In this prospective observational cohort study, we measured ABP with Welch‐Allyn 6100 ABP monitors at LA (< 1000 m) and HA (2,800‐3,000 m). Measured variables included heart rate and BP every 30 min while awake and every hour overnight, occurrence of BP ≥ 180/100 mmHg, sleep quality, nocturnal oxygen saturation (SpO2) and Lake Louise Score for acute mountain sickness (AMS). Results: Among 33 participants (mean age 57, 17 with underlying hypertension), 25 had LA and HA measurements. Average 24‐h MAP increased at HA (mean diff 6 mmHg, [95% CI: 2–10 mmHg], *p* = 0.04) due to elevated diurnal SBP. Diurnal SBP remained higher than LA after 72 h at HA. In participants without underlying hypertension, average 24‐h MAP rose from 81 mmHg to 90 mmHg at high altitude (mean diff 9 mmHg, [95% CI: 5–14 mmHg], *p* = 0.001), whereas 24‐h MAP did not increase at HA in those with underlying hypertension (mean diff 4 mmHg, [95% CI: ‐4 to 11 mmHg], *p* = 0.3). At HA, 24‐h MAP was similar in those without and with hypertension (mean diff 9 mmHg, [95% CI:0‐19 mmHg], *p* = 0.05). BP ≥ 180/100 mmHg was common at HA, but asymptomatic. Lower nocturnal SpO2 was associated with higher diurnal but not nocturnal SBP. There was no relation between 24h‐MAP, diurnal MAP or nocturnal MAP and AMS. Conclusion: In our tourist cohort, ABP increased at HA but was not clinically significant nor associated with any adverse short‐term outcomes.

**A016 INVESTIGATIONS ON THE PHYSIOLOGICAL AND INFLAMMATORY RESPONSES IN BALB/C MICE EXPOSED TO ACUTE HYPOXIA**

Mansi Balhara, R.J. Tirpude, Himadri Patir, M.P.K. Reddy

Defence Institute of Physiology and Allied Sciences, Defence Research Development Organisation, Timarpur, Delhi, India

Background: Ascending too fast or too high may cause the development of several high altitude related ailments in susceptible individuals due to low oxygen availability. High altitude hypoxia is known to induce an inflammatory response that may aggravate altitude ailments. CD44 is a cell surface glycoprotein reported to play an important role in many hypoxic conditions, viz., cancer hypoxia, activation‐induced hypoxia, and so forth. It is proposed as a potent therapeutic molecule in acute lung injury. We hypothesize that exposure to acute hypoxia may alter the expression of CD44 in the lung microenvironment that may contribute to the initiation and progression of lung pathologies at high altitude. The present study attempted to investigate the relevance of CD44 in altitude‐associated hypoxic conditions and its link to physiological and cellular responses. Materials and Methods: In this study, male BALB/c mice were subjected to extreme hypoxia at 8% FiO_2_ (~25000 feet) for 6, 24, and 48 h under normobaric hypoxic conditions. While vital physiological parameters such as Oxygen saturation (SpO_2_), Heart rate (HR), and Respiratory Rate (RR) were measured during exposure; CD44, oxidative stress parameters and hematological variables (Hemoglobin, Hematocrit, Platelets, NRBC), were determined after the stipulated time of each hypoxia exposure durations. Results: The results revealed a significant decrease (*p* < 0.0001) in SpO_2_ with increasing duration of hypoxia exposure as compared to normoxic conditions. Heart rate and Respiratory rate were also found to increase significantly. Furthermore, hypoxia led to elevated (*p* < 0.0001) oxidative stress and various hematological variables. Hypoxia and inflammation are mediated through several transcription regulators, especially HIF‐1α and NF‐κB. Conclusion: In this investigation, we observed marked upregulation of CD44 vis‐à‐vis HIF and NF‐κB in the lung homogenates of the hypoxic animals, indicating an interplay between hypoxia‐induced physiological and inflammatory responses. Preliminary findings indicate possible links between CD44 and hypoxia‐induced lung pathologies at high altitudes.

**A017 PROFILING OF CIRCULATING CELL‐FREE DNA AND ITS RELEASE MECHANISMS IN HIGH‐ALTITUDE PHYSIOLOGY AND PATHOPHYSIOLOGY**

Manzoor Ali^1,2^, Raushni Choudhary^1,2^, Krishna Kumar G.^1^, Kanika Singh^1,2^, Stanzen Rabyang^3^, Tashi Thinlas^3^, Aastha Mishra^1,2^

^1^Genomics and Genome Biology Unit, CSIR‐Institute of Genomics and Integrative Biology, Delhi, India

^2^Academy of Scientific and Innovative Research (AcSIR), Ghaziabad, India

^3^Department of Medicine, Sonam Norboo Memorial Hospital, Leh, India

Background: A high altitude (HA) is defined as an altitude more than 2500 m above sea level. Extreme conditions such as a decrease in barometric pressure with increased altitude lead to a reduction in the partial pressure of oxygen. Unacclimatized individuals may suffer from HA illnesses attributed to various genetic and epigenetic susceptibilities. Circulating cell free (*cf) DNA* are DNA fragments released in body fluids with a unique fragmentation pattern that can potentially be a diagnostic and prognostic biomarker in multiple diseases. Materials and Methods: In this study we have observed biological features and release mechanisms of cfDNA in the 3 study groups, healthy controls (*n* = 111), highlanders (HLs) (*n* = 20) and high‐altitude pulmonary edema (HAPE) patients (*n* = 90). HAPE patients were further segregated into mild (*n* = 30), moderate (*n* = 30), and severe (*n* = 30) categories. Results: The observations depicted significantly higher cfDNA concentrations in HAPE patients than controls (*p* < *0.001*). Among the HAPE patients, moderate and severe category exhibited 1.6‐fold and 3.62‐fold higher cfDNA concentrations than mild category respectively (*p* < *0.001*). The fragment length analysis of cfDNA indicated shorter cfDNA fragments in HAPE patients, reflected by a significantly lower cfDNA integrity index (*p* = *0.001*) in HAPE patients. The known mechanisms of cfDNA release are apoptosis, necrosis and active secretion. Hence, the study quantified markers like cytokeratin 18 (CK18) for total cell death, caspase‐cleaved cytokeratin 18 (cck18) for apoptosis and CD9 for active secretion. HAPE patients had significantly elevated levels of CK18 levels in the severe category (*p* < *0.001*). Furthermore, the cck18 levels were not significant among the three groups but interestingly a significant difference was observed in the severe category (*p* = *0.005*). The CD9 levels did not show any substantial difference in any study groups. Additionally, oxidative DNA damage analysis revealed significantly higher levels of 8‐OxodG in HAPE patients (*p* < *0.001*). Conclusion: Hence, the findings revealed that the severe HAPE patients had higher oxidative stress and their higher cfDNA levels were contributed by apoptotic pathways.

**A018 ALTERED CELLULAR ENERGETICS OF IMMUNE CELLS IN THE HYPOXIC NICHE OF HEPATOCELLULAR CARCINOMA**

Pushpa Yadav, Nirupama Trehanpati

Department of Molecular and Cellular Medicine, Institute of Liver and Biliary Sciences, New Delhi, India

Background: In the hypoxic tumor microenvironment (TME), cancer cells have ability to rewire metabolic pathways. Hypoxia can induce phenotypic changes in blood and liver with altered functionality which may promote their differentiation towards immunosuppressive phenotypes, leading to decreased production of pro‐inflammatory cytokines and impaired cytotoxic activity against tumor cells. However, in TME of hepatocellular carcinoma (HCC), functional immune cells and their metabolic modulations in this environment have not been adequately explored. Therefore, we aimed to analyse metabolic perturbations of immune cells in blood and TME of HCC. Materials and Methods: In this study, 27 end‐stage liver disease patients (12 HCC and 15 CLD) were included, and their isolated PBMCs from the blood and liver infiltrated lymphocytes (LILs) from liver tissues of patients were used for immune‐metabolic scan analysis using high dimensional flow cytometry. Results: HCC patients were significantly aged than CLD (*p* = 0.009) with higher AFP levels (*p* = 0.039) but no difference in PIVKA II levels. Although, albumin and AlT levels were increased in HCC (*p* < 0.05) but with decreased total bilirubin than CLD. Immune metabolic scan revealed that CLD and HCC patients had decreased total T cells and NKT in blood but with increased Lactate Dehydrogenase‐B (LDH‐B) (*p* < 0.05), 5′ AMP‐activated protein Kinase (AMPK) (*p* < 0.0001) and Hexokinase‐2 (HK2) (*p* = 0.0001) levels compared to healthy. These patients also showed increased NKp46+ NKT and CD158 + NKT cells compared to healthy. Both CD56 dim and CD56 bright NK cells expressed with higher LDH‐B, AMPK, NKp46 and CD158 in both end‐stage liver disease patients compared to healthy. However, HCC patients showed decreased activated NKT (defined as CD25 + NKT cells) but with high (*p* < 0.05) LDH‐B AMPK, NKp46, CD158, expression in HCC compared to CLD in blood. In TME of HCC, we observed that mTOR expressing T, NKT and activated NKT cells were increased with Nkp46. NKT and activated NKT cell compartments were decreased in patients with chronic liver disease and hepatocellular carcinoma. HK2, the first glycolysis enzyme, and AMPK, which regulates other key metabolic proteins, were increased. Conclusion: We conclude that to fulfill the metabolic demands of TME, NKT and activated NKT cells are positively regulated by mTOR leading to increased LDH‐B which is responsible for conversion of pyruvate to lactate.

**A019 CROSSTALK BETWEEN LUNG INTERSTITIAL MACROPHAGES IN HYPOXIC PULMONARY HYPERTENSION**

Rahul Kumar^1,2^, Aastha Mishra^3,4^, Qadar Pasha^5^, Brian B. Graham^1,2^

^1^Department of Medicine, University of California San Francisco, San Francisco, California USA

^2^Division of Pulmonary and Critical Care Medicine,

Zuckerberg San Francisco General Hospital, San Francisco, California, USA

^3^Genomics and Genome Biology Unit, CSIR‐Institute of Genomics and Integrative Biology, Delhi, India

^4^Academy of Scientific and Innovative Research (AcSIR), Ghaziabad, India

^5^Institute of Hypoxia Research, New Delhi, India

Background: Hypoxia‐induced pulmonary hypertension (PH) is a condition that results from exposure to high altitudes or pulmonary diseases. The interstitial pulmonary macrophages (IMs) are believed to contribute to developing this vascular pathology. While recent reports have characterized two major IM subsets‐ recruited CCR2+ IMs and resident FOLR2+ IMs‐ the mechanism by which these IMs interact with each other in hypoxia remains unknown. Results: Our study observed an increase in FOLR2+ resident interstitial macrophages in mice exposed to hypoxia. These macrophages expanded through proliferation and expressed the monocyte recruitment ligand CCL2. Additionally, we noted an increase in CCR2+ macrophages through recruitment, and these macrophages expressed the protein thrombospondin‐1, which functionally activates TGF‐beta and contributes to vascular disease. To investigate the role of monocyte recruitment in hypoxic PH, we blocked monocyte recruitment using either CCL2 neutralizing antibody treatment or CCR2 deficiency in the bone marrow compartment. Both approaches successfully suppressed hypoxic PH. To further support our findings, we analysed plasma samples from humans who traveled from low (225 m) to high (3500 m) elevations. We found an increase in thrombospondin‐1 and TGF‐β expression following ascent, which could be blocked by dexamethasone prophylaxis. In the hypoxic mouse model, dexamethasone prophylaxis replicated these findings by mechanistically suppressing CCL2 expression and CCR2+ monocyte recruitment. Conclusion: Overall, our data suggest a pathologic crosstalk between two distinct populations of interstitial macrophages, and targeting this crosstalk may have therapeutic implications for hypoxia‐induced PH.

**A020 INFLAMMATORY LUNG MICROENVIRONMENT IN LUNG CANCER‐ASSOCIATED PULMONARY HYPERTENSION**

Rajkumar Savai^1,2^

^1^Lung Microenvironmental Niche in Cancerogenesis, Institute for Lung Health, Justus Liebig University, Giessen D‐35392, Germany

^2^Department of Lung Development and Remodeling, Max‐Planck‐Institute for Heart and Lung Research, Bad Nauheim D‐61231, Germany

Background: Lung cancer (LC) is one of the major causes of mortality and morbidity worldwide. The increased prevalence of cardiovascular and pulmonary comorbidities in lung cancer patients contributes to exertional dyspnea, which is associated with poor prognosis. We recently provided evidence that pulmonary arterial (PA) enlargement, indicating pulmonary hypertension (PH), was observed in 250 of 519 patients with lung cancer. Results: Notably, 32 of 70 lung cancer patients had elevated pulmonary arterial systolic pressure (PASP) values on echocardiography. Extensive pulmonary vascular remodeling was consistently observed in histological analyses of human lung cancer tumor tissues. The cellular mechanism responsible for the association of lung vascular remodeling and PH in lung cancer involves chemokine release (IL8, CCL2, CCL5, GMCSF) from tumor cell–immune cell crosstalk, leading to perivascular accumulation of inflammatory cells. The clinical, histopathological, and experimental evidence from our study suggests a novel PH category associated with lung cancer. In lung cancer–associated pulmonary hypertension (LC‐PH), the hyperproliferative response in lung vascular cells driving remodeling and loss of lumen in lung vasculature is influenced not only by the tumor mass itself but also by the tumor microenvironment (TME). Innate immune cells predominate in the lung‐TME, with tumor‐associated macrophages (TAMs) playing a significant role in the pathogenesis of both LC and LC‐PH. The aberrant accumulation of TAMs in the TME depends on the differentiation of monocytes to macrophages, the activation of different macrophage phenotypes, and the interconversion within these phenotypes. Conclusion: TAMs induce malignancy in normal lung cells, such as pulmonary artery smooth muscle cells (PASMCs), and pulmonary artery adventitial fibroblasts (PAAFs), through various unexplored cellular and molecular mechanisms. TAMs may have distinct alterations in signal transduction pathways compared to PASMCs, PAAFs, and PAECs in LC‐PH, which could provide new directions for developmental therapeutics. In my presentation, I will discuss how lung microenvironmental cells (TAMs) influence the progression of LC and LC‐PH.

**A021 NUCLEOSOME FOOTPRINTS INFORM THE CONTRIBUTING CELL TYPES OF CELL‐FREE DNA MOLECULES IN HAPE**

Raushni Choudhary^1,2^, Manzoor Ali^1,2^, Krishna Kumar G.^1^, Stanzen Rabyang^3^,

Tashi Thinlas^3^, Aastha Mishra^1,2^

^1^Genomics and Genome Biology Unit, CSIR‐Institute of Genomics and Integrative Biology, Delhi, India

^2^Academy of Scientific and Innovative Research (AcSIR), Ghaziabad, India

^3^Department of Medicine, Sonam Norboo Memorial Hospital, Leh, India

Background: The fragmentation pattern in circulating cell‐free (cf) DNA reflects the position of the open chromatin regions (OCRs) of cells and tissue types contributing to the cfDNA release. Deep sequencing of the cfDNA enables fragment‐based analysis of genomic regions and can map the nucleosome occupancy and OCRs in plasma cfDNA. The nucleosomal organisation is highly specific across the cell types and correlates strongly with the gene regulatory processes. Through deep sequencing of cfDNA molecules, the highest read densities and coverage can be expected, which corresponds to the occurrence of the nucleosome‐depleted region (NDRs) in OCRs that aids in higher fragmentation of DNA sequences around that region, enabling identification of its cell types of origin. Extreme environmental conditions such as high altitude (HA) (altitude > 2500 m above sea level) can influence the nucleosomal landscape pattern derived from cfDNA in humans. Some sojourners visiting HA show better tolerance to hypoxia, but in some, a maladaptive response leads to High‐altitude pulmonary edema (HAPE), which is one of the acute and severe HA illnesses that develop upon rapid ascent to altitudes above 2500 m in otherwise healthy individuals. The quantitative analyses of differentially phased fragmented end signals of total cfDNA allow for the measurement of the relative contribution of tissues aiding the cfDNA pool under HA settings. Results: Deep sequencing and fragmentation analysis of circulating cfDNA fragments in the two study groups, HAPE patients and healthy controls, suggests the contribution of lymphoid and myeloid cells. In addition to this, we have also found contributions from other cells and tissues such as lung, liver, payers' patches, and spleen to the cfDNA pool under HA pathophysiology and physiology. Conclusion: Hence, cfDNA promises to be a minimally invasive biomarker that can map tissue injuries involved in the disease development and progression.

**A023 TIBETAN SPECIFIC GENETIC VARIANT PHD2** ^**D4E;C127S**^ **CONFERS HYPOXIA‐DEPENDENT PROTECTION AGAINST VIRAL INFECTIONS**

Riya Ghosh^1,2^, Garima Joshi^2^, Nishith M. Shrimali^2^, Kanchan Bhardwaj^1,2^, Tsewang Chorol^3^, Tashi Thinles^3^, Parvaiz Koul^4^, Josef T. Prchal^5^, Prasenjit Guchhait^2,^

^1^Manav Rachna International Institute of Research and Studies, Faridabad, India

^2^Regional Centre for Biotechnology, National Capital Region Biotech Science Cluster, Faridabad, India

^3^Sonam Nurboo Memorial Hospital, Leh‐Ladakh, Jammu and Kashmir, India

^4^Department of Internal and Pulmonary Medicine,

Sher‐i‐Kashmir Institute of Medical Sciences, Srinagar, India

^5^Department of Medicine, University of Utah School of Medicine and Huntsman Cancer Center and George E. Wahlin Veteran's Administration Medical Center, Salt Lake City, UT, USA

Background: At altitudes above 1500 meters, native Tibetan highlanders thrive without hypoxia‐related issues, attributed to a genetic variant PHD2^D4E;C127S^ in the *EGLN1* gene, present in approximately 80% of Tibetans, encodes the protein prolyl‐hydroxylase‐2 (PHD2). PHD2 regulates hypoxia‐inducible factor‐1α (HIF‐1α), crucial for cellular responses to low oxygen. The PHD2^D4E;C127S^ variant maintains HIF‐1α suppression even under hypoxic conditions by efficiently binding oxygen, preventing typical hypoxia‐induced effects like erythropoiesis and inflammation. We investigated the role of the PHD2^D4E;C127S^ variant on viral infections, focusing on its modulation of HIF1α levels and interferon (IFN) production under hypoxic conditions. Materials and Methods: *In vitro* studies using monocytes from individuals homozygous for the PHD2^D4E;C127S^ polymorphism and engineered cell lines (*EGLN1* ^c.[12C>G;380G>C]^) infected with Dengue virus‐2 (DENV2) and SARS‐CoV‐2 under both hypoxic (3% O_2_) and normoxic (21% O_2_) conditions. Results: Results demonstrated that PHD2^D4E;C127S^ monocytes exhibited reduced HIF1α levels and increased IRF‐3/7/9 and IFNα/β expression under hypoxia, conferring protection against DENV2 infection. Conversely, under normoxia, these cells showed elevated HIF1α levels, decreased IRFs and IFNα/β expression, and increased susceptibility to DENV2 infection. Similar patterns were observed with SARS‐CoV‐2 infection. Furthermore, treatment with HIF1α inhibitor CAY10585 enhanced IFNs and reduced viral infection in both in vitro and in vivo models. The gain‐of‐function PHD2^D4E;C127S^ variant was validated by demonstrating elevated IFN‐γ expression by CD4/CD8 T cells against SARS‐CoV‐2 RBD peptide under hypoxia in vitro. Conclusion: These findings elucidate a unique interplay between the PHD2^D4E;C127S^ variant and IFNs under different oxygen conditions, influencing viral infection susceptibility. The HIF1α inhibitor CAY10585 exhibits potential as a therapeutic agent against both dengue and SARS‐CoV‐2 by enhancing interferon production.

**A024 STATUS OF TELOMERE AND ITS RELATED GENES UNDER HYPOBARIC HYPOXIA OF HIGH‐ALTITUDE**

Rohit Kumar^1,2^, Manzoor Ali^2,3^, Swati Kumari^2,3^, Arun Paulose^1^,

Aastha Mishra^2,3^, Arpana Vibhuti^1^

^1^Department of Biotechnology, SRM University, Delhi‐NCR, Sonepat, Haryana, India

^2^Genomics and Genome Biology Unit, CSIR‐Institute of Genomics and Integrative Biology, Delhi, India

^3^Academy of Scientific and Innovative Research (AcSIR), Ghaziabad, India

Background: High‐ altitude (HA) is the region on the earth's surface above 2500 meters above sea level. The presence of decreased inspired partial pressure of oxygen (PaO_2_), and leads to decreased blood arterial oxygen saturation (SaO_2_) results in HA related disorders such as acute mountain sickness (AMS), high‐altitude pulmonary edema (HAPE), and high‐altitude cerebral edema (HACE). In addition, hypobaric‐hypoxia at HA may damage DNA due to the production of reactive molecular species and UV radiation. As a part of DNA, telomeres are the protective caps of the chromosomes with a dynamic, complex nature that maintains the integrity and length of chromosomal DNA. Several studies have shown the contribution of telomeres in the severity of HA disorders such as HAPE. Materials and Methods: Therefore, the present investigation aims to find the status of telomere length in the study groups consisting of the native people living at HA (highlanders or HLs), the sojourners, and the travelers from sea level to HA who are not affected (control) and affected by HAPE (HAPE patient). The telomere length has been compared in highlanders (*n* = 35), control (*n* = 64), and HAPE (*n* = 159). Results: The observation depicted significantly lower telomere length in HAPE patients compared to control and highlanders (*p* < 0.0001). However, the relative telomere length in the HAPE severity was mild (*n* = 41), moderate (*n* = 52), and severe (*n* = 51). The observation was not significant. The telomere length also depends on age, body mass index (BMI), and gender. After adjusting the age, BMI, and gender, the correlation study was done with the SpO_2_ level, respiration rate, pulse rate, and blood pressure to detect the presence and strength of a relationship between the study groups. Conclusion: Thus, the telomere length and the correlation investigation with its dependent and independent variables may suggest the unique role of telomere dynamics in HAPE and HA physiology.

**A025 VARIATION IN HYPOXEMIA ADAPTATION IN PATIENTS WITH PULMONARY ARTERIOVENOUS MALFORMATIONS‐ CLINICAL QUANTIFICATION AND PREDICTORS OF EXERCISE CAPACITY**

Shaarika Munshi, Sana Shah, Claire L. Shovlin

Department of Vascular Science, National Heart and Lung Institute, Faculty of Medicine, Imperial Center for Translational and Experimental Medicine, Imperial College London, London, UK

Background: Physiological responses to altitude‐induced hypoxemia can be modeled in patients with pulmonary arteriovenous malformations (PAVMs). These abnormal vascular connections between pulmonary arteries and veins result in chronic hypoxemia (oxygen saturations (SaO_2_) < 85%) as pulmonary arterial blood bypasses the pulmonary capillaries. Patients compensate through responses akin to altitude acclimatization, such as increasing hemoglobin production to maintain arterial oxygen content (CaO_2_), that is, SaO2(%)*Haemoglobin(g/L)*1.34/100. However, there are wide variations in patient exercise capacity for any given degree of hypoxemia. Hypothesizing that certain individuals demonstrate superior adaptive responses to others, this study aimed to investigate patient characteristics associated with higher exercise capacity. Materials and Methods: This was tested in a series of unselected, consecutive patients with PAVMs, and documented exercise capacity quantified by the validated Veterans Specific Activity Questionnaire (VSAQ), erect/supine SaO_2_ and pulse, hematological indices, and comorbidities/confounders status. Patient VSAQ scores (1 = lowest, 13 = highest) were converted to metabolic equivalents (METs) at maximal exercise capacity using 4.74+(0.97*VSAQ score)−(0.06*Age) [Myers, PMID:8147307]. Results: The 139 patients (mean age 49 years, 94 (67.6%) female) had METs ranging from 1.22 to 16.15 (mean 8.19) kcal/kg/min. Predictably, patients with comorbidities/confounders had lower METs. No relationship was observed with SaO_2_ and METs by univariate or multivariate regression. However, higher METs were observed in males, in patients with higher hemoglobin, higher CaO_2,_ and greater orthostatic pulse rise. In multivariate regression, 26% of the variation in patient exercise capacity (METs) was explained by a model comprising male sex (adjusted coefficient 2.17, *p* = 0.001), confounding disease (adjusted coefficient −2.00, *p* = 0.001) and either hemoglobin or CaO_2_, with a 0.03‐fold increase in METs for every 1 ml/L increase in CaO_2_ (*p* = 0.004) or 1 g/L increase in hemoglobin (*p* = 0.016). Conclusion: With 74% of variability in exercise capacity still unexplained, further work is examining genetic and earlier‐life variables.

**A026 PREGNANCY AND PULMONARY ARTERIAL HYPERTENSION: N‐TERMINAL PRO BRAIN NATRIURETIC PEPTIDE TRENDS AND OUTCOMES**

Shine Kumar^1^, Cini Sudhakara Prasad^2^, Sudha Sumathy^2^, Radhamony Kunjukutty^2^, Nitu Puthenveettil^3^, Amitabh Chanchal Sen^3^, Jeya Bawani Sivabalakrishnan^1^, Raman Krishna Kumar^1^

^1^Department of Pediatric Cardiology,

^2^Department of Obstetrics and Gynecology,

^3^Department of Anesthesiology, Amrita Hospital,

Amrita Vishwa Vidyapeetham University, Kochi, Kerala, India.

Background: Pulmonary Arterial Hypertension (PAH) poses significant risk to pregnancy with suboptimal maternal and fetal outcomes and is often contraindicated. Results: We identified 35 pregnancies in 22 women (mean age 27.9 ± 4.7 years, mean weight 50.6 ± 8.1 kg). The diagnoses were Eisenmenger syndrome (16, 72.7%), postoperative residual PAH (3, 13.6%), idiopathic pulmonary arterial hypertension (2, 9.1%) and one (4.5%) had systemic lupus erythematosus. Twenty three babies (65.7%) were born alive, gestational age of 35.1 ± 2.9 weeks, 47.8% at term, with a birth weight of 2.1 ± 0.8 kg. There was elevation of NT‐proBNP in the initial 72 h post‐delivery (median 138 pg/mL, IQR: 112–561). Those with a persistent rise beyond 72 h (median 686 pg/mL IQR: 370–3691) had prolonged recovery postpartum (median post‐delivery hospital stay 18 days, IQR: 12–22) reflecting continued right ventricular stress and maladaptation. There was single maternal mortality (4.5%). Conclusion: Maternal and fetal outcomes of pregnancy associated with PAH are better with strict surveillance and multidisciplinary team effort. Postpartum period remains the most vulnerable time. NT‐proBNP trends during this period may be a promising objective monitoring tool in identifying at‐risk subsets, thus improving outcomes.

**A027 THE GENOTYPIC VARIATIONS AND PENETRANCE OF INHERITED CARDIAC CHANNELOPATHIES IN THE INDIAN POPULATION**

Shraddha Handa^1,2^, Sridhar Sivasubbu^3^, Nitin Rai^4^, Nitish Naik^4^, Aastha Mishra^1,2^

^1^Genomics and Genome Biology Unit, CSIR‐Institute of Genomics and Integrative Biology, Delhi, India

^2^Academy of Scientific and Innovative Research (AcSIR), Ghaziabad, India

^3^Vishwanath Cancer Care Foundation, Mumbai, India.

^4^Department of Cardiology, All India Institute of Medical Sciences, New Delhi, India

Background: Cardiac channelopathies are a group of clinical arrhythmogenic disorders that affect the heart's electric system, due to mutations in genes encoding for these ion channels. Genetic mutations affecting various ion channels lead to inherited cardiac arrhythmias, increasing the susceptibility of affected individuals to sudden cardiac death. The most common cardiac channelopathies include Long QT Syndrome, Brugada Syndrome, Catecholaminergic Polymorphic Ventricular Tachycardia, and Short QT Syndrome. Each condition has distinct genetic mutations and clinical manifestations, but all share a probability of causing life‐threatening arrhythmias, particularly in young and otherwise healthy individuals. This prospective study aims to elucidate the genotypic variations and their penetrance in the Indian population and to evaluate the effectiveness of genotype and phenotype aided relationships in these patients. Materials and Methods: Over the reporting period, samples were collected from two nodal centers, AIIMS New Delhi and SCTIMST Trivandrum, including probands and their family members. Genomic DNA isolation and whole exome sequencing were performed on the probands; variants were classified according to ACMG‐AMP guidelines into pathogenic, likely pathogenic, benign, likely benign, and variants of uncertain significance. Results: The findings indicate a significant correlation between specific genetic mutations and clinical phenotypes, aiding in risk stratification and management of cardiac channelopathies. Pinpointing these specific genomic alterations causing the disease can offer additional clinical insights. To validate these variants and to study the inheritance and co‐segregational pattern, the Sanger sequencing technique was opted. Conclusion: Future research will encompass further validation of findings and expansion of the genetic analysis to additional samples, along with a potential for developing low‐cost, effective screening techniques, improving patient outcomes and reducing the incidence of sudden cardiac death in the Indian population.

**A028 NON‐OCCUPATIONAL ANTHRACOFIBROSIS/ANTHRACOSILICOSIS IN LADAKH**

Sonam Spalgais

Pulmonary Medicine, Vallabhbhai Patel Chest Institute, University of Delhi, Delhi, India

Background: Anthracosis is caused by inhalation and deposition of black pigments (carbon) and is commonly seen in coal workers. Anthracosis with narrowing of bronchial wall is anthracofibrosis. Air pollution, biomass smoke, and smoking are also causes. Silicosis is caused by the inhalation, retention, and reaction to silica. Both diseases are mostly due to occupational exposure. Nonoccupational anthracofibrosis and anthracosilicosis are reported from Ladakh. Ladakh is one of the world's highest inhabited plateaus at > 10,000 feet above sea level. This altitude and geography lead to long duration of winter (4–5 months) with low temperatures, resulting in exposure to smoke and soot from the burning biomass fuel. Dust storms are also common here, containing fine dust particles, and most people are exposed to them, especially during spring. Clinically, these diseases are difficult to differentiate from other diseases like COPD, TB and cancer due to similar clinico‐radiological presentation. Bronchoscopy is an important tool for confirming anthracosis pigment deposition, and bronchial biopsy to rule out other causes like malignancy. This leads to unnecessary repeated invasive procedures in such patients. Among Ladakhi, the disease is commonly seen in elderly females and non‐smokers with biomass fuel history. Cough, breathlessness and sputum are common symptoms. On radiology, mass‐like lesions with nodules are a common finding; consolidation, fibrotic bands and collapse are also seen. Even a case of recurrent effusion due to pleural anthracosis was also reported from Ladakh. On bronchoscopy, presence of anthracotic pigment with destruction of bronchial wall in bilateral lung with multilobar involvement is common. Conclusion: Awareness about environmental hygiene, installation of chimneys and exhaust fans, and use of non‐combustive substances are important measures. A systemic study also needs to find the role of silica and soot in preventing this irreversible disease.

**A029 HYPOXIA‐MEDIATED TRANSLATIONAL DYNAMICS: NEW DISCOVERIES AND PERSPECTIVES**

Soni S. Pulamsetti

Max‐Planck‐Institute for Heart and Lung Research, Bad Nauheim D‐61231, Germany

Background: Hypoxia, a condition characterized by insufficient oxygen levels, plays a crucial role in various pathological events in the lung (pulmonary hypertension, pulmonary fibrosis, COPD and lung cancer). Measurements of translation efficiency and steady‐state mRNA levels by RNA sequencing have shown that reprogramming of translation efficiency is an independent and more important factor in hypoxic protein induction than changes in mRNA levels. My presentation will focus on the translational remodeling that occurs during acute and prolonged hypoxia exposure of pulmonary vascular cells. Specifically, I will address (i) translational plasticity, in which cells employ unique mechanisms in response to a broad spectrum of biological changes during acute and prolonged hypoxia, (ii) how the translational regulatory proteins are regulated and how they orchestrate a hypoxia‐activated switch from cap‐dependent to cap‐independent mRNA translation that promotes selective mRNA translation and increased survival of pulmonary vascular cells, (iii) what are the specific features of the hypoxic translation system that require its activation? and (iv) the possible contributions of noncoding RNAs to hypoxic translational remodeling will be discussed.

**A030 ANEMIA AT HIGH ALTITUDE ‐ REAL OR A CALCULATION ERROR?**

Spalchen Gonbo^1^, Padma Angmo^2^

^1^Community Health Centre Khaltsi, Health Department, Leh, India

^2^Integrated Child Development Scheme, Leh, India

Background: As per the NFHS‐5 (2019–20) data, anemia prevalence in the under 5 years of age population in UT Ladakh is 92.5%, the highest in the country. It means that every 9 out of 10 children in Ladakh are anemic. As per observation, anemia prevalence is high in Ladakh as is in the rest of the country, but 92.5% seems exaggerated. It is possible that it is due to the use of a correction factor to compensate for the increase in Hb in response to hypoxia. Studies have shown that responses to hypoxia in terms of increased Hb levels differ in different populations and are less prevalent in children. As such, a uniform correction factor may not apply to all. The correction factor may not apply at all in certain populations, like the ethnic population in the Himalayas. All studies on the prevalence of anemia at high altitudes, including one done at Leh in 2013 (Medical Check for Children) by a medical group from the Netherlands, used a correction to compensate for the increase in Hb in response to hypoxia. The calculation is based on the method devised by CDC Atlanta, based on a study on high‐altitude populations in the Andes. As per various studies, the response to hypoxia is different in different ethnic groups. The Tibetan population is found to respond differently and may not respond with increased Hb. Similarly, the response is also different in various age groups, and as such, it has been found that the degree of Hb increase in response to hypoxia is lesser in children. Materials and Methods: As per a cross‐sectional study of Hb level on 897 children (6 months to 5 yrs.) attending various ICDS Centers of (Anganwadi) all over Leh district done between Nov 2023 to May 2024, Hb level was calculated randomly in all children (Well babies) attending Anganwadi center using Hb meter approved by WHO (reflectance photometry for Methemoglobin). Children were categorized as anemic and non‐anemic. Therapeutic measures were done as per the Anemia Mukth Bharat program. Children with severe anemia were referred to SNM Hospital for further tests. Results: As per this cross‐sectional study, anemia prevalence (any anemia) was 15%, with the WHO cutoff taking < 11 gm% as anemia. On applying correction for high altitude, it rose to 83%. As a clinician practicing at Leh we don't use any correction factor and take the Hb reading as such. This is as per personal experience after anemia workups and physical examinations. Conclusion: Studies have observed Tibetans' lower average hemoglobin concentration relates to higher physical work capacity and probably lowers the risk of thrombosis, chronic mountain sickness, or pre‐eclampsia than visitors from low altitudes. Further studies with larger samples may strengthen the hypothesis.

**A031 STROKE AT HIGH ALTITUDE: CURRENT PERSPECTIVES**

Sunil K. Munshi

Nottingham University Hospitals NHS Trust, Nottingham, UK

Background: Stroke at high altitude (HA) is less common after short term visits to HA than High Altitude Cerebral Edema, Acute Mountain Sickness and high altitude Acute Mountain Sickness. Much of the data has come from studies from Northern India (Kashmir, Ladakh), China, Andes, Saudi Arabia, Switzerland, and the US. The definition of HA is different in the Indian literature as compared to the western literature. Various metanalyses provide insights into the pooled prevalence of stroke in high‐altitude areas, highlighting variations based on geographic regions, different altitudes, duration of stay and sampling type. Deep venous pulmonary and cerebral venous thrombosis have been reported after exposure to HA. Stroke due to cerebral infarction is commoner than cerebral hemorrhage. Cerebral venous thrombosis is also common and presents with a different constellation of symptoms. Apart from routine risk factors of stroke (hypertension, smoking, diabetes, obesity, atrial fibrillation) several other mechanisms account for stroke at HA–namely polycythemia, hyperviscosity, enforced sedentariness, poor water quality with dehydration, hypoxia leading to hypercoagulability, increased fibrinogen, Factor VIII, d‐Dimers, thrombopoietin, erythropoietin, Platelet Factor IV, platelet adhesiveness, endothelial damage, transient arrhythmias and alcohol. Quite often, younger people are affected and the infarcts are large. Conclusion: Preventive strategies are vital and should include screening individuals for underlying cardiovascular disease and neurovascular disorders, prevention of dehydration and regular mobilization and education. As the causes are multi‐factorial, and the management varies, clinicians should familiarize themselves with strategies to detect and prevent cerebrovascular events with appropriate investigations in all individuals embarking on these high‐risk adventures.

**A032 THE ROLE OF REMOTE ISCHEMIC CONDITIONING IN IMPROVING THE DISEASE RELATED MARKERS IN INTERSTITIAL LUNG DISEASE**

Swati Kumari^1,2^, Rohit Kumar^1^, Vishal Bansal^3^, Aastha Mishra^1,2^

^1^Genomics and Genome Biology Unit, CSIR– Institute of Genomics and Integrative Biology, Delhi, India

^2^Academy of Scientific and Innovative Research (AcSIR), Ghaziabad, India

^3^Department of Physiology, Vallabhbhai Patel Chest Institute, University of Delhi, Delhi, India

Background: Hypoxia plays a crucial role in the pathophysiology of a majority of pulmonary diseases. A normal level of oxygen is indispensable for the proper functioning of the body, and a level lower than this could manifest pulmonary diseases such as interstitial lung disease (ILD). A reduced oxygenation capacity and pulmonary fibrosis (PF) compromise lung function in ILD, which has a poor prognosis and limited treatment options. The current pharmacological treatments for ILD are limited and have multiple side effects. A few nonpharmacological treatments have proven effective in cardiovascular complications by improving cardiovascular function, oxygenation capacity, and overall pulmonary function. One such treatment is remote ischemic conditioning (RIC), which is a noninvasive endogenous therapeutic strategy in which short nonlethal episodes of alternating ischemia and reperfusion applied on a remote organ before or after a subsequent prolonged episode of ischemic insult renders the target organ protected from such insults in the future. Materials and Methods: In this study, the effect of RIC is observed in an in vivo mouse model created by prolonged diesel exhaust (DE) exposure that mimics the pathology of idiopathic pulmonary fibrosis (IPF), which is one of the types of ILD. Although the study design includes multiple animal groups, here we present the data for the three groups: control (C), DE‐induced PF model (DE), and DE‐induced PF model receiving RIC during PF‐induction (per) (DE + RIC). Results: The histopathological assessment showed a less pronounced severity in DE + RIC than in the DE group. Additionally, there is a significant increase in weight of mice in the DE + RIC group, followed by the C and DE groups (*p* < 0.05). There are also improvements in the disease‐related and inflammatory markers in DE + RIC compared to DE. Moreover, the assessment of mitochondrial functioning parameters showed a similar pattern. Conclusion: Thus, the results suggest a role of RIC in mitigating the pathological damage associated with IPF in our in vivo model, which warrants further in‐depth investigations.

**A033 ANDEAN VERSUS TIBETAN ADAPTIVE MODES: EVIDENCE AT REST AND DURING EXERCISE**

Taylor S. Harman^1^, Pontus K. Holmström^2^, Kelsey C. Jorgensen^3^, Anne Kalker^4^, Melisa Kiyamu^5^, Kimberly T. Zhu^3^, Trevor A. Day^6^, Abigail W. Bigham^3^, Tom D. Brutsaert^7^

^1^ Department of Anthropology, Syracuse University, NY, USA

^2^Department of Health Sciences, Mid‐Sweden University, Sundsvall, Sweden

^3^Department of Anthropology, University of California, Los Angeles, CA, USA

^4^Department of Anesthesiology, Raboud Medical Center, Nijmegen, Netherlands

^5^Departamento de Ciencias Biológicas y Fisiológicas, Universidad Peruana Cayetano Heredia, Lima, Peru

^6^ Department of Biology, Mount Royal University, Calgary, Alberta, Canada

^7^Department of Exercise Science, Syracuse University, Syracuse, NY, USA

Background: Indigenous high‐altitude populations exhibit physiological adaptations to environmental hypoxia. It has been hypothesized that two of these populations, Andeans and Tibetans, demonstrate distinct adaptive modes, with the former characterized by increased blood oxygen content and the latter characterized by increased blood flow. Materials and Methods: To investigate this hypothesis, we recruited two groups of healthy adults (ages 18‐35 y) with highland ancestry who were born and currently reside at high altitude. The groups were: Andean Quechuas recruited in Cerro de Pasco, Peru (AND, *n* = 301) and Tibetan Sherpas recruited in Pheriche, Nepal (SHP, *n* = 64). Participants were tested in field laboratories using identical equipment and protocols at nearly identical altitudes (4330 m and 4371 m, respectively). We assessed various physiological variables at rest, submaximal, and maximal exercise. Results: We found that although some phenotypes aligned with the above hypothesis, the majority did not. For example, as predicted, AND displayed significantly higher hemoglobin concentrations than SHP (*p* < 0.001). However, we failed to replicate the previously published finding that hemoglobin concentration was correlated with maximal aerobic capacity (VO_2_ max). Further, the AND and SHP groups displayed no significant differences in VO_2_max. We found that the SHP group had higher minute ventilation, ventilatory equivalents for oxygen and carbon dioxide, and arterial oxygen saturation at lower submaximal workloads. However, this trend reversed at maximal exercise, during which the AND group exhibited significantly higher values for each variable (*p* < 0.01). Conclusion: These results suggest that the adaptive modes of these two populations are more complex than asserted by the hypothesis described above.

**A034 SEXUAL DIMORPHISMS AND THE ROLE OF ESTROGEN RECEPTOR ALPHA SIGNALING IN CARDIOPULMONARY ADAPTATION TO CHRONIC DEVELOPMENTAL HYPOXIA: NOVEL INSIGHTS FROM PHYSIOLOGIC AND GENETIC STUDIES**

Tim Lahm

National Jewish Health, University of Colorado, Colorado, USA

Background: Humans living at high‐altitude (HA) have adapted to this environment by increasing pulmonary vascular and alveolar growth. RNA sequencing data from a novel murine model that mimics this phenotypical response to HA suggested estrogen signaling via estrogen receptor alpha (ERα) may be involved in this adaptation. We hypothesized that ERα is a key mediator in the cardiopulmonary adaption to chronic hypoxia and sought to delineate the mechanistic role ERα contributes to this process by exposing novel loss‐of‐function ERα mutant (ERαMut) rats to simulated HA. Materials and Methods: ERα mutant or wild type (WT) rats were exposed to normoxia or hypoxia starting at conception and continued postnatally until 6 weeks of age. Results: Both WT and ERαMut animals born and raised in hypoxia exhibited lower body mass and higher hematocrits, total alveolar volumes (Va), diffusion capacities of carbon monoxide (DLCO), pulmonary arteriole (PA) wall thickness, and Fulton indices than normoxia animals. Right ventricle adaptation was maintained in the hypoxia setting. While no major physiologic differences were seen between WT and ERαMut animals at either exposure, ERαMut animals exhibited smaller mean linear intercepts and increased PA total and lumen areas. Hypoxia exposure or ERα loss‐of‐function did not affect lung mRNA abundance of vascular endothelial growth factor, angiopoietin 2 or apelin. Conclusion: Sexual dimorphisms were noted in PA wall thickness and PA lumen area in ERαMut rats. While ERα loss‐of‐function affected alveolar size and PA remodeling, ERα's role in the cardiopulmonary physiologic adaptation to simulated HA appears inconsistent for the time point and degree of hypoxia studied.

**A035 QUERCETIN PROPHYLAXIS: AN APPROACH TO ALLEVIATE HIGH ALTITUDE ILLNESS**

Vaishnavi Rathi, Sarada S.K. Sagi

Defence Institute of Physiology and Allied Sciences, Defence Research Development Organisation, Timarpur, Delhi, India

Background: Hypoxia emerges as the major barrier at high altitude for military troops and mountaineers. Prophylaxis with carbonic anhydrase inhibitors like acetazolamide (AZD) has been suggested for better adaptation and survival at altitude more than 2500 m. However, due to innumerable adverse effects of AZD like paraesthesias, polyuria, fatigue, nausea and dyspnea, there is an urgent need for a highly efficient molecule that can work against the maladies caused by hypobaric hypoxia at high altitude regions. Materials and Methods: Hence, the study presented here assesses the prophylactic efficacy of quercetin in preventing hypoxia‐induced complications at a height of 7620 m (25,000 ft.) for 12 h in male SD rats. This study elucidates a comparative aspect between quercetin and AZD to mitigate the alterations occurring under hypobaric hypoxic stress. Male SD rats were supplemented orally with quercetin (50 mg/Kg BW) and AZD (50 mg/Kg BW) and then subjected to hypobaric hypoxia. The results obtained suggested that hypoxia‐induced oxidative imbalance (ROS, Protein carboxylation) and antioxidant inactivity (GR and catalase) were significantly restored by supplementation of quercetin in the plasma of rats (*p* < 0.001). However, AZD‐supplemented normoxia rats showed enhanced levels of antioxidants in the plasma of these rats in comparison with the normoxia control group. The prophylactic potential of both drugs was further evaluated with hematological (RBC, WBC and Platelets) and blood gas parameters (PO2, PCO2 and SPO2). Western blot analysis studies determined the differential expressions of Nrf‐2 and HO‐1 in rat kidney homogenates and revealed quercetin's better efficacy in modulating these proteins (*p* < 0.001) under hypoxia compared to the control group. Results: Supplementation of AZD under normoxia conditions significantly altered these proteins in comparison to normoxia control group. Acetazolamide is a well‐known carbonic anhydrase inhibitor and thus brings about these changes by initiating metabolic acidosis in the rats. Quercetin, a natural phytoflavonoid showed unmodified changes in rats supplemented with quercetin under normoxia. Conclusion: These results indicate that, quercetin is a safe and potent drug for high altitude acclimatization.

**A036 EXAMINING THE INFLUENCE OF YOGA ON PULSE RATE, OXYGEN SATURATION, AND HYPOXIC EFFECTS: ANALYSIS OF PSYCHOPHYSIOLOGICAL PARAMETERS AND ANAEROBIC METABOLISM SHIFT**

Varun Malhotra, Avinash Thakare, Rajay Bharshankar, Shweta Mishra, Naveen Ravi

Department of Physiology, AYUSH, All India Institute of Medical Sciences, Bhopal, India

Background: *Pranayamic* breathing, involving continuous inhalation, breath retention, and exhalation, aims to convert venous blood into oxygenated blood. However, the impact of deep breathing, which involves large oxygen intake, on oxygen saturation and anaerobic metabolism during Yoga remains unclear. This study examines the effects of Yoga on pulse rate, oxygen saturation, and psycho‐physiological parameters. Materials and Methods: Fifty‐two subjects aged 15–70 performed a “Yoga module for the Healthy Heart” for 45 min at AYUSH, All India Institute of Medical Sciences, Bhopal. Pulse rate and oxygen saturation were measured before and after yoga using a pulse oximeter. Additionally, a pilot study was conducted on ten regular yogic practitioners using the DINAMIKA HRV instrument to assess psycho‐physiological parameters before and after a 35–40 min yogic routine. Results: Results showed a significant decrease in pulse rate (81.98 ± 13.05 to 74.98 ± 11.64, *p* < 0.0001), indicating parasympathetic dominance shift. Oxygen saturation decreased marginally (97.40 ± 1.11 to 97.21 ± 1.30), suggesting anaerobic metabolism during yoga. Psycho‐physiological parameters were significantly affected post‐yoga, including stress index, power, vegetative index, regulation, neurohumoral regulation, psycho‐emotional state, energy resources, complex index, harmonization, and biological age. Furthermore, through the power of will, yogic practitioners could draw cosmic energy into the spine, aiding its renewal. Conclusion: Yoga facilitated mental focus amidst physical distractions, inducing relaxation by calming heart rate, respiration, and circulation.

**A037 INTERVENTIONS TO IMPROVE HYPOXIA TOLERANCE**

Vishal Bansal

Department of Physiology, Cardiopulmonary Rehabilitation Unit, Vallabhbhai Patel Chest Institute, University of Delhi, Delhi, India

Background: Rapid ascent to high altitude is associated with decreased aerobic exercise capacity due to decreased arterial oxygen content and limitation in maximal cardiac output. A significant portion of impairment in exercise tolerance has been attributed to hypoxic pulmonary vasoconstriction (HPV). It has been proposed that the underlying mechanisms responsible for HPV are largely mediated through vasoactive and inflammatory pathways contributing to hypoxia intolerance. Several pharmacological and nonpharmacological interventions to improve hypoxia tolerance attenuate HPV at simulated and high altitude environments and improve arterial oxygen saturation (SpO2), however, inconstant results of these interventions have been observed on physical performance. Recently, ischemic preconditioning (IPC) has been shown to induce systemic effects that protect the myocardium and other organs from ischemic injury. These effects have also been observed in pulmonary vasculature, where it has been demonstrated that the hypoxic increase in pulmonary artery systolic pressure during acute simulated altitude conditions is significantly attenuated by IPC, thereby, improving hypoxia tolerance. The protective effects of IPC on local and remote tissues are largely attributed to effects on vasoactive and inflammatory pathways. Interestingly, IPC and HPV have similar mechanistic pathways, that is, hypoxia, yet confer opposing effects. Exercise training imparted under Pulmonary Rehabilitation program is being increasingly used in the management of patients with acute and chronic cardio‐respiratory diseases where it has been clearly demonstrated to reduce dyspnea and improve exercise performance. Meta‐analyses and systemic reviews have shown that beneficial effects of exercise training are mediated by reduction in inflammatory and oxidative stress markers, improvement of endothelial function, increased oxidative capacity and skeletal muscle mass, enhanced vagal and lower sympathetic tone in chronic heart and respiratory patients which overall improves hypoxia tolerance. Results: Our research (unpublished data) has shown a trend towards attenuation of post‐hypoxia exposure increase in B‐type Natriuretic Peptide by IPC along with exercise training. Conclusion: This indicates cardio‐protection during hypoxia is being conferred by these interventions.

## Data Availability

Data sharing not applicable to this article as no datasets were generated or analysed during the current study.

